# Early Childhood in Marginalized Roma Communities: Health Risks and Health Outcomes

**DOI:** 10.3389/ijph.2024.1606784

**Published:** 2024-03-22

**Authors:** Daniela Filakovska Bobakova, Zuzana Dankulincova Veselska

**Affiliations:** ^1^ Department of Health Psychology and Research Methodology, Faculty of Medicine, P. J. Safarik University, Kosice, Slovakia; ^2^ Olomouc University Social Health Institute, Palacky University in Olomouc, Olomouc, Czech Republic

**Keywords:** health, health risks, early childhood, poverty, marginalized Roma communities

## Abstract

**Objectives:**

This study aims to compare selected early childhood health risks and health outcomes of children from marginalized Roma communities (MRCs) in Slovakia with those of the majority.

**Methods:**

We obtained cross-sectional data from mother-child dyads from the majority (N = 109) and MRCs (N = 143) via questionnaires and from medical records. Socioeconomic status, health risks and health outcomes were compared using chi-square and Mann-Whitney U tests in SPSS.

**Results:**

Mothers from MRCs reported significantly worse socioeconomic status. Air quality in the households in MRCs was significantly worse, affected by heating with stoves, burning fresh wood and indoor smoking. The diet composition of children from MRCs was characterized by shorter breastfeeding and unhealthy diet composition less fresh fruits and vegetables, more processed meat products, and sweet and salty snacks. Children from MRCs more often suffered from respiratory and diarrheal diseases, used antibiotics and were hospitalized.

**Conclusion:**

The health and healthy development of children living in MRCs is endangered by various poverty-related factors. Persistent differences in exposures and health in early childhood should be a priority goal of the state’s social and health policies.

## Introduction

Child poverty threatens the health and wellbeing of millions of children worldwide and its eradication remains a challenge even in developed and rich countries [1]. Early childhood health and development are an important basis for future health and wellbeing in adulthood [2]. Poverty negatively affects children from conception onwards—from survival to their health, nutrition, and cognitive development [3]. In Slovakia, the most disadvantaged children endangered by poverty are those growing up in marginalized Roma communities (MRCs) [4].

Early childhood health and development in MRCs is endangered by poor quality of housing, indoor air pollution and absence of running water and sewerage [4]. The environment of MRCs is often infested with pollutants and parasites due to the presence of industrial objects, illegal landfills [5], and unwormed animals [6]. Lack of resources in poor families results in food insecurity and insufficient nutrition [7, 8].

The abovementioned factors negatively affect the health and healthy development of children. Living in MRCs is associated with poorer health regardless of socioeconomic differences within the community [9]. Children from MRCs suffer from infectious and chronic diseases, injuries, poisoning and burns more often than children from the majority population and make up the majority of hospitalized children [10]. Especially alarming is the infant mortality rate, which is 2.5 times higher in children from MRCs compared to the general population and even exceeds the neonatal mortality rate, which is rather unusual as neonatal mortality rates usually exceed infant mortality rates [11].

Poverty, segregation, environmental risks, higher rates of morbidity and malnutrition are contextual characteristics that threaten healthy development in early childhood [3, 12] and children from MRCs facing multiple problems are thus in a disadvantaged starting position compared to their better-off peers. Experiencing poverty in early childhood affects healthy development significantly worse than if it is experienced later in life [13] as persistent poverty has a cumulative effect on children’s development [14] and long-term consequences for health [3, 12].

This study aims to compare selected early childhood health risks and health outcomes of children from (MRCs) in Slovakia with those of the majority.

## Methods

### Sample and Procedure

Data from the first wave of the longitudinal RomaREACH (Research on Early Childhood in marginalized Roma communities) study were used to explore differences between children from MRCs and the majority population. Data collection took place in the Presov and the Kosice regions in 2021–2022. These regions hold the highest share of the marginalized Roma population [4]. Mother-child dyads with complete data on the domains of interest from marginalized Roma communities (N = 143) and the Slovak majority population (N = 109) with children aged 12–21 months were included. Mothers of the children were approached to participate in the study via primary paediatricians during regular preventive check-ups. Mothers from MRCs were also recruited via Roma health mediators and social workers directly in the communities and mothers from the majority via parental groups on social media. After signing informed consent, mothers filled in the questionnaire independently or with the assistance of the researchers if the assistance was needed due to limited literacy. In the subsample which entered the study via cooperation with primary paediatricians (N = 158), data from the health records were retrieved.

### Measures

A series of sociodemographic variables, health risks and selected health outcomes were assessed in mothers and their children from MRCs and the majority.

Belonging to a *marginalized Roma community* was assessed based on the Atlas of Roma Communities - the sociographic mapping conducted by the Office of the Government Plenipotentiary for the Roma Communities in cooperation with the Institute for Work and Family Research undertaken in 2019 [4]. For verification purposes, the following question was asked: “Are your closest neighbours mostly Roma?”

*Background variables* included the child’s sex and age in months, number of siblings maternal age and marital status.

To assess the level of *maternal education* [15] we used the question: “What education have you completed? (Elementary school, Apprentice school, Secondary school, University).” We categorized the education of mothers into three categories: 1. Elementary education, 2. Secondary education including apprentice school, and 3. University education.

*Billing problems* [9] were assessed by asking whether the household has encountered problems paying for some of the expenses in the past year: 1. Rent; 2. Water, electricity, gas; 3. Food and clothing; 4. Repayments of loans; and 5. Healthcare costs. The response categories were Yes/No.

*Insufficient house equipment* [9] was assessed by asking about the availability of amenities in the household: 1. Cold running water; 2. Hot running water; 3. Working flushing toilet; 4. Working bathroom or shower; and 5. Electricity. The response categories were Yes/No.

*SES-related stress/worries* comprised questions about experiencing stress and/or worries frequently related to low socio-economic status in the past 3 months: 1. Loss of housing (concerns about losing a roof over your head); 2. Nothing to eat (fear that there will be nothing to eat in the household); 3. Removal of children (fear that your children will be taken away from you); 4. Money for heating (concerns about the cold and lack of money for heating); 5. Loss of job (worries about losing your job or someone close to you); 6. Conflicts/fights in the neighbourhood (stress from fights or sharp conflicts with people outside the household). The response categories were Yes/No.

Number of *persons per room* indicating overcrowdedness was calculated as the number of household members (i.e., adults living in the household and children living in the household) divided by the number of living rooms and bedrooms.

To assess the risk resulting from *contact with unvaccinated animals* we asked whether the respondents regularly come into contact with animals (cats, dogs, etc.) that are not regularly checked by a veterinarian (dewormed and vaccinated). The response categories were Yes/No.

*Air quality in the household* was assessed by asking about heating methods, and the type of solid fuel used in the household, with interest in those heating with a stove and burning fresh wood. Indoor smoking was assessed by asking whether smoking is done inside the house. The response categories were Yes/No.

*Daily smoking during pregnancy* was assessed by asking about smoking during pregnancy with the following response categories: No, I did not smoke/I smoked occasionally, but not daily/I smoked daily/I used to smoke, but gradually during pregnancy I stopped. We categorized the answers into two categories: 1. Daily smoking during pregnancy and 2. Other.

*Food consumption of children* [9] was assessed by asking how many times during the past week the child ate: 1. Apple, banana, orange or other fresh fruit; 2. Tomato, sweet pepper, salad or other fresh vegetables; 3. Milk, yoghurt, cheese or other dairy products; 4. Salami, sausage, ham or other meat products; 5. Schnitzel, roast chicken, stewed meat or other meat; 6. Pasta, dumplings, pancakes or other flour dishes; 7. Coke, soft drinks or other sweetened drinks; 8. Candies, chocolate cookies, chocolates or other sweets (ordinary biscuit does not count); 9. Chips, peanut crisps and other salty snacks (pure corn crisps do not count). The response categories were Not at all/Once or twice/Several times.

*Breastfeeding* was assessed by asking about the number of months of breastfeeding and whether the child is still breastfed.

*Health complaints of children in the past month* as reported by mothers were assessed by asking whether the child suffered from any of the following problems in the last month: 1. Headache; 2. Stomach ache; 3. Cold; 4. Flu; 5. Cough; 6. Constipation; 7. Diarrhea; 8. Sadness/tearfulness; 9. Irritability/bad mood; 10. Fatigue; 11. Sleeplessness; 12. Loss of appetite.

*The number of respiratory, diarrheal, and febrile diseases, antibiotic use and hospitalizations since birth* was based on the children’s medical records.

### Statistical Analyses

First, we described the sample using descriptive statistics. Next, differences between the two groups (mothers from MRCs vs. mothers from the majority population) were assessed using the Mann–Whitney *U*-test and Chi-squared test. Statistical analyses were performed using IBM SPSS 23 for Windows.

## Results

### Demographic and Socioeconomic Description of the Sample

Table 1 shows the descriptive statistics for mothers from MRCs and mothers from the majority population. Significant differences between the groups were found in most of the explored measures of interest. Mothers from MRCs were significantly younger, less educated, had more children and lived rather in partnership than in marriage as opposed to the mothers from the majority. Households in MRCs had more billing problems (except for problems with paying for rent) and were insufficiently equipped with cold and hot running water, flushing toilets, shower and electricity. Mothers from MRCs also reported significantly more SES-related stress and worries compared to mothers from the majority (except for worries about losing their jobs).

**TABLE 1:** Demographic and socioeconomic description of the sample–comparison between majority and marginalized Roma communities (Research on Early Childhood in marginalized Roma communities study, Slovakia, 2021–2022).

	Majority		MRCs		Total		*p*-value
	N = 109		N = 143		N = 252		
	Mean (SD)		Mean (SD)		Mean (SD)		
Age of mothers in years	32.10 (4.22)		25.20 (6.48)		28.18 (6.57)		0.000
Age of children in months	15.93 (1.08)		15.80 (1.55)		15.86 (1.37)		0.344
Number of siblings	0.63 (0.75)		2.97 (2.76)		1.96 (2.43)		0.000
Persons per room	1.16 (0.39)		4.40 (3.02)		3.00 (2.80)		0.000

	N	(%)	N	(%)	N	(%)	*p*-value
Child’s sex							0.850
Boys	49	45.0	66	46.2	115	45.6	
Girls	60	55.0	77	53.8	137	54.4	
Marital status							0.000
Single	7	6.4	17	11.9	24	9.5	
Married	88	80.7	51	35.7	139	55.2	
Divorced	0	0	2	1.4	2	0.8	
In partnership	14	12.8	73	51.0	87	34.5	
Widow	0	0	0	0	0	0	
Education mother							0.000
Elementary	2	1.8	112	78.3	114	45.2	
Secondary	23	21.1	31	21.7	54	21.4	
University	84	77.1	0	0	84	33.3	
Education father (N = 237)							0.000
Elementary	1	0.9	102	78.5	103	43.6	
Secondary	45	42.5	28	21.5	73	30.9	
University	60	56.6	0	0	60	25.4	
Billing problems							
Rental	2	1.8	10	7.0	12	4.8	0.057
Water, electricity, gas	4	3.7	25	17.5	29	11.5	0.001
Food, clothing	3	2.8	39	27.3	42	16.7	0.000
Repayments of loans	1	0.9	15	10.5	16	6.3	0.002
Healthcare costs	2	1.8	42	29.4	44	17.5	0.000
Insufficient house equipment							
No cold running water	0	0	66	46.2	66	26.2	0.000
No hot running water	0	0	87	60.8	87	34.5	0.000
No flushing toilet	0	0	91	63.6	91	36.1	0.000
No shower/bath	0	0	90	62.9	90	35.7	0.000
No electricity	0	0	9	6.3	9	3.6	0.008
SES-related stress/worries^a^							
Loss of housing	3	2.8	28	21.1	31	12.8	0.000
Nothing to eat	2	1.8	35	26.3	37	15.3	0.000
Removal of children	0	0	20	15.0	20	8.3	0.000
Money for heating	1	0.9	38	28.6	39	16.1	0.000
Loss of job	15	13.8	31	23.3	46	19.0	0.060
Conflicts/fights in the neighbourhood	0	0	17	12.8	17	7.0	0.000

^a^ 10 missing cases.

### Health Risks

Table 2 shows the differences in selected health risks between mothers from MRCs and mothers from the majority population. Significant differences between the groups were found in most of the health risk indicators. Contact with unvaccinated animals and smoking during pregnancy was more common in MRCs. Air quality in households in MRCs was significantly more often affected by heating with stoves, burning fresh wood and indoor smoking. The diet composition of children from MRCs was characterized by shorter breastfeeding, less frequent consumption of fresh fruits and vegetables and more frequent consumption of processed meat products, sweetened drinks, sweets, and salty snacks when compared with children from the majority population.

**TABLE 2:** Health risks–comparison between majority and marginalized Roma communities (Research on Early Childhood in marginalized Roma communities study, Slovakia, 2021–2022).

	Majority		MRCs		Total		*p*-value
	N = 109		N = 143		N = 252		
	N	(%)	N	(%)	N	(%)	
Contact with unvaccinated animals^a^	6	5.7	58	41.1	64	26.0	0.000
Air quality in the household							
Heating with a stove	16	14.7	126	88.1	142	56.3	0.000
Burning fresh wood	4	3.7	72	50.3	76	30.2	0.000
Indoor smoking^b^	0	0	67	47.2	67	26.7	0.000
Daily smoking during pregnancy^c^	2	1.8	52	37.1	54	21.7	0.000
Food consumption of children in past week^b^							
Fresh fruits							0.012
Not at all	0	0	4	2.8	4	1.6	
Once or twice	10	9.2	28	19.7	38	15.1	
Three times/more	99	90.8	110	77.5	209	83.3	
Fresh vegetables							0.001
Not at all	12	11.0	43	30.3	55	21.9	
Once or twice	35	32.1	37	26.1	72	28.7	
Three times/more	62	56.9	62	43.7	124	49.4	
Dairy products							0.112
Not at all	3	2.8	7	4.9	10	4.0	
Once or twice	8	7.3	21	14.8	29	11.6	
Three times/more	92	89.9	114	80.3	212	84.5	
Processed meat products							0.000
Not at all	31	28.4	22	15.5	53	21.1	
Once or twice	41	37.6	29	20.4	70	27.9	
Three times/more	37	33.9	91	64.1	128	51.0	
Cooked meat dishes							0.436
Not at all	11	10.1	22	15.5	33	13.1	
Once or twice	37	33.9	43	30.3	80	31.9	
Three times/more	61	56.0	77	54.2	138	55.0	
Flour dishes							0.445
Not at all	5	4.6	12	8.5	17	6.8	
Once or twice	35	32.1	47	33.1	82	32.7	
Three times/more	69	63.3	83	58.5	152	60.6	
Sweetened drinks							0.000
Not at all	102	93.6	49	34.5	151	60.2	
Once or twice	3	2.8	32	22.5	35	13.9	
Three times/more	4	3.7	61	43.0	65	25.9	
Sweets							0.000
Not at all	68	62.4	13	9.2	81	32.3	
Once or twice	31	28.4	34	23.9	65	25.9	
Three times/more	10	9.2	95	66.9	105	41.8	
Salty snacks							0.000
Not at all	89	81.7	43	30.3	132	52.6	
Once or twice	15	13.8	30	21.1	45	17.9	
Three times/more	5	4.6	69	48.6	74	29.5	
Breastfeeding							
Still breastfeeding	52	47.7	42	29.4	94	37.3	0.003
	Mean (SD)		Mean (SD)		Mean (SD)		
Number of months of breastfeeding	10.99 (5.97)		6.69 (7.12)		8.61 (6.96)		0.000

^a^ 6 missing cases.

^b^ 1 missing cases.

^c^ 3 missing cases.

### Health Outcomes of Children

The results presented in Table 3 revealed significant differences between children from MRCs and the majority population in various health complaints reported by mothers. Children from MRCs suffered from headaches, stomach aches, flu, cough, diarrhoea, sadness or tearfulness, fatigue and sleeplessness significantly more often than children from the majority. Although children from MRCs also suffered from cold, irritability and loss of appetite more often than children from the majority, these differences were not statistically significant.

**TABLE 3:** Health complaints of children in the past month reported by mothers–comparison between majority and marginalized Roma communities (Research on Early Childhood in marginalized Roma communities study, Slovakia, 2021–2022).

	Majority		MRCs		Total		*p*-value
	N = 109		N = 143		N = 252		
	N	(%)	N	(%)	N	(%)	
Headache	1	0.9	21	14.7	22	8.7	0.000
Stomach ache	4	3.7	52	36.4	56	22.2	0.000
Cold	55	50.5	87	60.8	142	56.3	0.100
Flu	7	6.4	40	28.0	47	18.7	0.000
Cough	36	33.0	87	60.8	123	48.8	0.000
Constipation	7	6.4	7	4.9	14	5.6	0.600
Diarrhoea	14	12.8	44	30.8	58	23.0	0.001
Sadness, tearfulness	7	6.4	46	32.2	53	21.0	0.000
Irritability	26	23.9	49	34.3	75	29.8	0.073
Fatigue	8	7.3	30	21.0	38	15.1	0.003
Sleeplessness	11	10.1	34	23.8	45	17.9	0.005
Loss of appetite	16	14.7	30	21.0	46	18.3	0.200

Based on the children’s medical records shown in Table 4, a significantly higher number of respiratory and diarrheal diseases, as well as numbers of antibiotic use and hospitalizations since birth were found in children from MRCs compared to children from the majority. The difference in the number of febrile diseases was not statistically significant.

**TABLE 4:** The number of respiratory, diarrheal, and febrile diseases, antibiotic use and hospitalizations since birth based on the children’s medical records–a comparison between majority and marginalized Roma communities (Research on Early Childhood in marginalized Roma communities study, Slovakia, 2021–2022).

	Majority	MRCs	Total	*p*-value
	N = 93	N = 65	N = 158	
	Mean (SD)	Mean (SD)	Mean (SD)	
Respiratory diseases	2.08 (2.51)	3.98 (4.08)	2.86 (3.37)	0.001
Diarrheal diseases	0.35 (0.70)	1.00 (1.51)	0.62 (1.15)	0.006
Febrile diseases	1.23 (1.45)	2.09 (2.72)	1.58 (2.10)	0.121
Use of antibiotics	0.77 (1.24)	2.32 (2.93)	1.41 (2.32)	0.000
Hospitalization^a^	0.32 (0.79)	0.87 (1.42)	0.55 (1.12)	0.002

^a^ 5 missing cases.

## Discussion

Children from MRCs included in our study grow up in significantly worse socioeconomic situations compared to their better-off peers. Households in MRCs often lack running water or flushing toilets, and face billing problems and various worries related to low socio-economic status. The health of these children is more often endangered by contact with unvaccinated and unwormed animals, poor quality of air in the household or inadequate diet composition. Also, the health outcomes of children from MRCs are worse compared with children from the majority.

### Socioeconomic Deprivation

We found significant differences between MRCs and the majority in many indicators of socioeconomic deprivation. More than ¾ of parents from MRCs have only elementary education in contrast to the majority population where the vast majority of parents have at least secondary education. More than ¼ of households from MRCs encountered problems paying common expenses such as food, clothing, and healthcare costs. Almost half of the households in MRCs do not have running water and more than half of households are missing hot water or flushing toilets. In terms of education or water supply, these results are comparable to the results of the HepaMeta study conducted more than 10 years ago in the same area [9]. In other indicators, e.g., paying common expenses such as food or clothing and healthcare costs the situation seems to be worse compared to the past results [9]. Also compared to the EU SILC MRK survey conducted in 2020 on a sample of 1,000 households in 70 towns/villages collecting data for assessing the fulfilment of the indicators of the new EU Strategic Framework for Roma Equality, Inclusion, and Participation the educational level of our sample is comparable, but the share of households without water or flushing toilets is higher in our sample [16]. These differences might indicate that our study included more segregated poorer communities, locations with unapproved housing and fewer better-off communities with rental housing. SES-related feelings of stress and worries were reported significantly more often by mothers from MRCs. This points to the degree to which everyday struggles of people living in poverty, such as worries related to loss of housing, food insecurity, removal of children, lack of money for heating and conflicts in the neighbourhood, translate into feelings of stress. Socioeconomic disadvantage and ethnic minority status were previously shown to be associated with an increased level of stress [17, 18], which, together with related racism, discrimination and segregation, negatively influence children’s health and development [19].

### Health Risks

According to our results, early childhood health in MRCs is threatened by various factors that are significantly more prevalent there than in the majority population. Mothers from MRCs reported contact with unvaccinated and undewormed animals significantly more often than mothers from the majority. Within severe socio-economic disadvantage of MRCs it is very problematic to maintain hygienic standards. Such conditions are known to be associated with greater contamination of soil samples with parasites, and greater number of dogs infected with parasites. As a consequence, there is a greater prevalence of parasites in children [6, 20]. Air quality in the households in MRCs might be according to our results affected by more frequent use of stoves for heating, burning fresh wood and indoor smoking. These factors are significantly more prevalent in MRCs compared to the majority where stoves are used only in a small percentage of households and burning fresh wood or indoor smoking is very unlikely. This is in line with findings by Majdan et al. [21], who reported increased Carbon Monoxide (CO), Carbon Dioxide (CO_2_) and total particulate matter (PM) in Roma settlements in Slovakia suggesting that indoor air pollution has the potential to be a health threat.

Our results indicate, that children from MRCs have less adequate diet composition compared to children from the majority. Although they do not seem to differ in the frequency of consumption of dairy products, cooked meat dishes and flour dishes they eat significantly less fresh fruits and vegetables. Consumption of sweetened drinks, sweets and salty snacks is much more common in MRCs compared to the majority. Similar results could be found in the adult population included in the HepaMeta study [8] where consumption of fruits and vegetables was less frequent and consumption of soft drinks more frequent in MRCs in comparison with the majority population. Also, more recent data from the EU SILC MRK study reported a higher percentage of those not able to afford quality food in comparison to the majority population [16]. In addition to diet, almost half of children from the majority population are still breastfed, but only less than a third of children from MRCs. The average duration of breastfeeding in MRCs is approximately 4 months shorter than the duration of breastfeeding of children from the majority. Differences between Roma and non-Roma mothers in breastfeeding are not always present in the literature. The study by Stamenkovic et al. [22] in Serbia revealed that the prevalence of exclusive breastfeeding was almost the same among mothers in both non-Roma and Roma populations. However, they did find out, that in the Roma subsample, exclusive breastfeeding was less likely in mothers from urban areas and with the lower socioeconomic status, which is a situation comparable to Roma mothers from MRCs in our study. A qualitative study by Herrero et al. [23] reported that barriers that limited and largely hindered breastfeeding in the group of Roma mothers included lack of confidence, lack of family support, and low participation in maternal education activities.

### Health Outcomes of Children

Health outcomes of children from MRCs were found to be worse than those of children from the majority. Health complaints of children reported by mothers related to respiratory (flu, cough) and diarrheal (stomach ache, diarrhoea) diseases were reported significantly more frequently by mothers from MRCs compared to mothers from the majority. Moreover, children from MRCs were more often reported to be sad or tearful and to suffer from headaches, fatigue or sleeplessness. The study by Dostal, Topinka and Sram [24] similarly reported that at the age of 0–2 years, the incidence of influenza, intestinal infectious diseases and viral diseases was significantly higher in Roma than in non-Roma children. It could be assumed that headaches can be related to poorer water availability, respiratory diseases to hygiene and poorer air quality, and diarrheal diseases to parasites and poorer water quality which are in our study more likely in MRCs. Also based on health records, the occurrence of respiratory and diarrheal diseases is more frequent in children from MRCs than in children from the majority. The most alarming is the frequent use of antibiotics as children from MRCs used antibiotics on average more than twice since birth which is more than twice compared to the children from the majority. Hospitalizations are more common among these children as well. These results are in line with previous studies conducted in Slovakia [10] and also in Serbia [25]. The study by Djurovic et al. [25] reported that the length of hospitalization of Roma children was significantly higher, as well as the number of laboratory tests performed and number of drugs prescribed. First, children in MRCs are more likely to be ill, and their medical conditions are so severe that antibiotics are used more often, and they require hospitalization more often. However, another interpretation is also possible, which indicates that Roma children are treated differently with lower affordability and availability of healthcare, leading to worsening of their health status and resulting in finding care later on in the worse state, which already requires antibiotics and/or hospitalizations due to more complications [25].

### Strengths and Limitations

Our study provides evidence of persisting early childhood inequities between children from MRCs and the majority in a variety of health risk indicators and demonstrates health disparities in data reported by mothers as well as primary care providers. It includes the most vulnerable children in the age that bears the highest importance for future health and development. Some limitations need to be mentioned. The cross-sectional design does not allow conclusions about causality. The description of multiple risk factors does not cover the complexity of disadvantages that threaten early childhood health and development in MRCs nor provide evidence for the interplay of these factors and how they contribute to health outcomes. Furthermore, self-reported data are prone to social desirability, which may cause an underestimation of the prevalence of health risks.

### Implications for Practice, Policy and Future Research

Combating child poverty is one of the sustainable development goals [1]. Poverty and unhealthy living conditions of children from MRCs need to be addressed on community as well as structural levels. Raising the educational level of parents would increase their ability to participate in the labour market and provide a nurturing environment for their children. However, it is also essential to fight against discrimination against Roma in the labour market and create suitable job opportunities and opportunities to improve qualifications and work skills to enable the social mobility of parents living in poverty [26]. Various measures to achieve these and many other goals, such as improving access to drinking water or healthcare, have been part of strategies for Roma integration since the early 2000s [27, 28], but our findings indicate insufficient success of the efforts so far. Non-profit organizations have taken over the role of the state in many areas, but often, even if they are relatively successful, they focus on only one of the problem areas such as the self-help construction of houses [29] or supporting the psychomotor development of children in early childhood [30]. However, due to the interconnectedness and scope of the problems that require attention, a much more holistic approach is needed. Increasing health literacy should also be part of such an approach as inappropriate diet composition of children for which parents are responsible at this age might be influenced not only by limited resources but also by unhealthy choices (e.g., excessive consumption of sweets and sweetened drinks or shorter duration of breastfeeding of children). Educational opportunities for mothers could thus contain informal community learning targeting health-promoting behaviours such as breastfeeding and nutrition. Therefore, continuous and targeted support of health mediation directly in communities, such as that implemented by the contribution organization of the Ministry of Health - Healthy Regions [26], should be guaranteed by the state.

Further longitudinal research should explore the roles of modifiable factors in causal pathways between health risks and health outcomes and focus on the development and evaluation of successful intervention programs to combat early childhood inequities.

### Conclusion

Poverty and unhealthy living conditions of children from MRCs included in our study are fundamentally worse compared to children from the majority. The health and healthy development of children living in overcrowded households without running water is threatened by many factors such as poor sanitary conditions, quality of air or food insecurity. Parents experiencing financial hardship struggle to provide their children with clothing and regular food intake of adequate quality. Children experience various health complaints, use antibiotics more often and are hospitalized more often than children from the majority. Ensuring equal chances for health and healthy development for children from MRCs will require a concentrated effort, therefore these persistent differences in exposures and health in early childhood should be a priority goal of the state’s social and health policies.
